# Sex differences in leucocyte telomere length in a free‐living mammal

**DOI:** 10.1111/mec.13992

**Published:** 2017-01-27

**Authors:** Rebecca L. Watson, Ellen J. Bird, Sarah Underwood, Rachael V. Wilbourn, Jennifer Fairlie, Kathryn Watt, Eliane Salvo‐Chirnside, Jill G. Pilkington, Josephine M. Pemberton, Tom N. McNeilly, Hannah Froy, Daniel H. Nussey

**Affiliations:** ^1^ Institute of Evolutionary BiologyAshworth LaboratoriesUniversity of EdinburghThe King's BuildingsCharlotte Auerbach RoadEdinburghEH9 3FLUK; ^2^ SynthSysUniversity of EdinburghThe King's Buildings, Waddington BuildingMax Bourne CrescentEdinburghEH9 3BFUK; ^3^ Moredun Research InstitutePentlands Science ParkBush LoanMidlothianEH26 0PZUK

**Keywords:** granulocyte, lymphocyte, *Ovis aries*, polygyny, sexual selection, Soay sheep

## Abstract

Mounting evidence suggests that average telomere length reflects previous stress and predicts subsequent survival across vertebrate species. In humans, leucocyte telomere length (LTL) is consistently shorter during adulthood in males than in females, although the causes of this sex difference and its generality to other mammals remain unknown. Here, we measured LTL in a cross‐sectional sample of free‐living Soay sheep and found shorter telomeres in males than in females in later adulthood (>3 years of age), but not in early life. This observation was not related to sex differences in growth or parasite burden, but we did find evidence for reduced LTL associated with increased horn growth in early life in males. Variation in LTL was independent of variation in the proportions of different leucocyte cell types, which are known to differ in telomere length. Our results provide the first evidence of sex differences in LTL from a wild mammal, but longitudinal studies are now required to determine whether telomere attrition rates or selective disappearance are responsible for these observed differences.

## Abstract 2

see also the Perspective by Dantzer and Garratt

## Introduction

Telomeres are G‐rich repeat sequences of DNA at the ends of chromosomes, which form complexes with proteins that protect linear chromosomes from DNA repair machinery and the so‐called end replication problem (Blackburn 1991; De Lange 2004). Telomeres shorten with each cell replication and are also highly sensitive to damage by reactive oxygen species (von Zglinicki 2002), but can be replenished through the action of the enzyme telomerase (Armanios & Blackburn 2012). When telomere length drops below a critical threshold, it triggers the onset of cellular senescence, and thus, there may be close links between telomere attrition and cellular ageing (Hemann *et al*. 2001; Gomes *et al*. 2011). Average telomere length (TL), which is typically measured from DNA obtained from whole blood samples, has been shown to decline with age in numerous species and is a biomarker of considerable current interest in human epidemiology and evolutionary ecology (Monaghan & Haussmann 2006; Blackburn *et al*. 2015). In humans, TL in adulthood appears predictive of late‐onset diseases and, in some studies, mortality risk (Cawthon *et al*. 2003; Blackburn *et al*. 2015; Rode *et al*. 2015), whilst prior experience of chronic stress is associated with shorter TL (Shalev 2012; Monaghan 2014). Studies of wild birds and mammals have also recently found associations between short TL and reduced subsequent survival or recapture rates (Bize *et al*. 2009; Barrett *et al*. 2013; Fairlie *et al*. 2016).

In humans and laboratory rodents, females generally have longer TL than males in adulthood (Barrett & Richardson 2011; Gardner *et al*. 2014). This pattern mirrors the pervasive gender difference in longevity observed in humans and many other mammals, leading to speculation that sex differences in TL may be related to differences in lifespan (Stindl 2004; Aviv *et al*. 2005). A recent meta‐analysis of forty adult human data sets concluded that females have significantly longer telomeres than males (Gardner *et al*. 2014), and more recent large‐scale studies have confirmed this pattern (Lapham *et al*. 2015; Berglund *et al*. 2016). However, it remains unclear whether sex differences in TL are present in early life or emerge in later adulthood due to differences in telomere attrition rates in humans. Several studies have failed to document significant sex differences in TL measured in umbilical cord blood (Okuda *et al*. 2002; Akkad *et al*. 2006; Aubert *et al*. 2012; Shi‐Ni *et al*. 2013), although a recent larger‐scale study did find significantly longer telomeres in newborn females than males (Factor‐Litvak *et al*. 2016). Studies of captive primates have yielded equivocal results: no sex differences in TL were found in cynomolgus macaques (*Macaca fascicularis*; Gardner *et al*. 2007), whilst female rhesus macaques (*Macaca mulatta*) had significantly longer TL than males in leucocytes but not in other tissue types (Smith *et al*. 2011). In laboratory populations of rats (*Rattus rattus*; Cherif *et al*. 2003; Tarry‐Adkins *et al*. 2006) and Algerian mice (*Mus spretus*; Coviello‐McLaughlin & Prowse 1997), females were also reported to have longer telomeres than males. To date, studies of sex differences in TL in nonhuman mammals remain limited to primates and rodents in captivity: we could find only one study comparing TL between the sexes in a wild mammal population. This study of European badgers, which show strong sexual size dimorphism, found no evidence of sex differences in mean TL or changes in TL with age (*Meles meles*; Beirne *et al*. 2014).

The pattern of sex differences in TL across vertebrate species appears variable, and several nonmutually exclusive explanations have been proposed for this variation, including heterogametic disadvantage and differences in the effects of sex hormones on oxidative stress and telomerase function (Barrett & Richardson 2011; Gardner *et al*. 2014). It has also been hypothesized that, in species with sexual size dimorphism, increased cell proliferation rates and oxidative stress associated with increased growth in the larger sex could reduce TL (Stindl 2004; Barrett & Richardson 2011). Furthermore, increased parasite burdens in males relative to females have been documented in polygynous mammals and proposed as a driver of male‐biased mortality (Moore & Wilson 2002). Experiments in laboratory‐kept house mice (*Mus musculus*) demonstrated faster leucocyte TL loss in males than in females following repeated bacterial infection, raising the possibility that sex differences in TL could reflect sex differences in the response to infection (Ilmonen *et al*. 2008). Polygynous males also typically exhibit secondary sexual characteristics other than increased body size (e.g. seasonal coloration, vocalizations, ornaments and weapons) and highly energetically expensive intrasexual competition for access to mates (Andersson 1994). Male investment in these reproductive traits may be associated with increased cellular proliferation or oxidative stress, resulting in faster TL attrition in males relative to females (Monaghan 2010; Barrett & Richardson 2011). A recent review found little consistent evidence that sex differences in TL were associated with heterogamety, the degree of body size dimorphism or mating system (Barrett & Richardson 2011). However, direct tests for sex‐specific associations between TL and either weight, reproductive investment or parasite burden within polygynous species remain rare (Olsson *et al*. 2011; Beirne *et al*. 2014).

In mammals, which have enucleated red blood cells, TL measured in DNA extracted from blood samples reflects the average leucocyte telomere length (LTL). This is in contrast to other vertebrate groups with nucleated erythrocytes, where it is erythrocyte telomere length (ETL) that is predominantly measured in blood. This represents a challenge for mammalian studies, as LTL encompasses a range of different white blood cell types which have different functions and roles within the immune system and show differences in proliferative capacity, telomerase expression and ultimately telomere length (Weng 2001). For instance, in humans and baboons, granulocytes have longer telomeres than lymphocytes in adulthood, most likely due to the fact that granulocytes are terminally differentiated cells whilst lymphocytes have the capacity to rapidly replicate and differentiate (Baerlocher *et al*. 2007; Kimura *et al*. 2010; Aubert *et al*. 2012). Lymphocytes also vary in telomere length, with naïve T cells having longer telomere lengths in comparison with memory T cells, again due to greater proliferative history of the latter (Weng 2001; Aubert *et al*. 2012). The composition of circulating leucocyte cell types can change profoundly with age and vary between sexes (Linton & Dorshkind 2004; Pawelec *et al*. 2010; Giefing‐Kröll *et al*. 2015), and changes found in average LTL in relation to age and sex could therefore reflect changes in underlying cell population structure (Weng 2001). However, studies in humans and primates have reported very strong within‐individual correlations in TL measured in different leucocyte subpopulations and among different tissues (Baerlocher *et al*. 2007; Gardner *et al*. 2007; Kimura *et al*. 2010; Aubert *et al*. 2012; Daniali *et al*. 2013). Based on this apparent ‘synchrony’ in TL across tissue and cell types, it has been argued that among‐individual variation in LTL reflects differences in the TL of the haematopoietic stem cell pool, which is primarily determined genetically and by early life environment (Daniali *et al*. 2013). However, studies investigating the dependence of LTL and its associations with age, sex and other traits on variation in leucocyte population structure in nonprimate mammals are currently lacking.

Here, we tested sex differences in LTL in a free‐living population of Soay sheep on the St Kilda archipelago. Soay sheep have a polygynous breeding system: males compete for mating opportunities with oestrous females during the autumn rut and have highly skewed reproductive success, with a handful of males obtaining the majority of paternities each year (Clutton‐Brock & Pemberton 2004). As is typical in polygynous systems, males are larger and shorter lived than females: by 5 years of age, males average around 38 kg and females around 24 kg in summer with maximum recorded lifespan of 10 years in males and 16 years in females (Clutton‐Brock & Pemberton 2004). This population is parasitized by a variety of strongyle gastrointestinal nematodes, and parasite burdens – as estimated by faecal egg counts (FEC) – are predictive of overwinter survival and greater in males than in females at all ages (Wilson *et al*. 2004; Hayward *et al*. 2009). Horns, which are grown incrementally each year, are an important secondary sexual trait in males in Soay sheep and horn length is positively associated with subsequent male annual reproductive success (Johnston *et al*. 2013). Both sexes exhibit normal and greatly reduced (scurred or polled) horn growth phenotypes, and the genes underpinning this polymorphism have recently been identified (Johnston *et al*. 2013). We have recently shown that LTL is positively associated with survival in early life in female Soay sheep (Fairlie *et al*. 2016) and that age‐related variation in the proportion of different T‐cell subtypes is present in this population (Nussey *et al*. 2012). However, we have yet to test for sex differences in LTL or examine whether and how LTL is associated with variation in leucocyte cell structure in the population. Here, we examine whether sex differences in LTL are present and whether evident sex differences are dependent on age, weight or strongyle FEC, as well as testing the hypothesis that costly investment in male horn growth should be reflected by reduced LTL. We also measure the proportions of different leucocyte cell types which are known to differ in telomere dynamics and test whether variation in leucocyte population structure can explain observed associations among LTL, sex, age and investment in secondary sexual characteristics.

## Materials and methods

### Study system and sample preparation

The Soay sheep is a primitive breed of domestic sheep that has been living on the remote St Kilda archipelago with minimal human management for the last few millennia. Since 1985, the sheep living in the Village Bay area of the main island in the archipelago, Hirta, have been the subject of individual‐based study (Clutton‐Brock & Pemberton 2004). As part of this study, individuals are caught and tagged within a few days of birth in spring. An individual's horn type is identified at capture as follows: ‘normal’ horns are sturdy and consist of a bony core covered in a keratin sheath, whilst ‘scurred’ horns consist of keratin but lack a bony core. A ‘polled’ phenotype is present only in females and involves a complete absence of visible horn growth. Around 85% of males and 35% of females in the population have normal horns (Clutton‐Brock & Pemberton 2004). Each August, 50–60% of the resident population are caught in temporary corral traps. At capture, blood and faecal samples are taken and each animal is weighed to the nearest 0.1 kg and horn length is measured from the base of the horn, along the outer curvature to the tip, with each annual growth increment noted (Clutton‐Brock & Pemberton 2004). This study uses data and samples from animals caught during the Augusts of 2014 (78 males and 174 females) and 2015 (66 males and 174 females).

Two 9‐mL lithium heparin Vacuettes of blood were taken from each individual and kept in a cool box or fridge from the point of sampling until further processing within 24 h of sampling. The first Vacuette of blood was spun at 1008 ***g*** for 10 min and the plasma layer was then drawn off and replaced by the same quantity of 0.9% NaCl solution and spun again at 1008 ***g*** for 10 min. The intermediate buffy coat layer, comprising mainly white blood cells, was then drawn off into a 1.5‐mL Eppendorf tube and stored at −20 °C until used to assay leucocyte telomere length. Faecal samples were available for 427 of the captured individuals, and strongyle and Strongyloides FEC were estimated in these samples using a modified McMaster technique (following Gulland & Fox 1992).

### Leucocyte cell measurements

Within 12 h of collection, 5 μL of whole blood from the second Vacuette was applied to one end of a standard glass microscope slide. The drop of blood was then spread with the edge of a second slide at a 45° angle to produce an even film. Slides were air‐dried overnight and stained using a Quick‐Diff Kit stain (Gentaur, London) the following day, as per the manufacturer's instructions. Differential white blood cell counts were conducted back in the laboratory in Edinburgh. Briefly, 100 cells were counted at 40× magnification using the ‘battlement track’ method and based on staining and morphology, identified as either lymphocytes, eosinophils or neutrophils (Bain 2014). Basophils and monocytes were observed too rarely to analyse. From this data, we calculated the granulocyte (neutrophils and eosinophils)‐to‐lymphocyte ratio (GLR) and this ratio was used in subsequent analyses. Only slides with a clear regular monolayer of cells were counted, and slides with uneven cell density or unclear staining were omitted, leaving 465 GLR measurements available for subsequent analyses. See Watson *et al*. (2016) for information on repeatability of these measurements.

A further 1 mL of whole blood from the second Vacuette was used to prepare a formalin‐fixed sample of lymphocytes, which was stored at 4 °C until subsequent flow cytometry analysis back in Edinburgh (following Nussey *et al*. 2012). We were able to estimate the proportions of lymphocytes that were helper T cells (CD4+), cytotoxic T cells (CD8+) and within each of these cell types the proportion that were putatively naïve cells (CD45RA+). Full methodological details are presented in the online supplementary material. From this data, we calculated the CD4 T‐cell‐to‐CD8 T‐cell ratio as well as the proportions of helper T cells and cytotoxic T cells that were naïve, and we used these three measurements in subsequent analyses.

### Telomere length measurement

Genomic DNA was extracted from buffy coat using the Qiagen DNeasy Blood and Tissue Kit following manufacturer's guidelines for animal blood (Cat# 69581, Manchester, UK). The protocol was modified slightly to facilitate sample flow through the spin columns which subsequently improved DNA yield and purity (see online supplementary material). Following DNA extraction and elution in buffer AE (10 mm Tris‐Cl, 0.5 EDTA, pH 9.0), a strict quality control protocol was implemented to determine DNA quality and integrity. First, each sample was individually tested for DNA yield and purity using a NanoDrop ND‐1000 9 spectrophotometer (Thermo Scientific, Wilmington DE, USA). Samples yielding <20 ng/μL were immediately rejected. Samples yielding ≥20 ng/μL were checked for DNA purity; acceptable ranges for absorption were 1.7–2.0 for 260/280 nm ratio and 1.8–2.2 for 260/230 nm ratio. Acceptable samples were then diluted to 10 ng/μL and their DNA integrity was assessed by running 20 μL (200 ng total DNA) on a 0.5% agarose gel. Samples were scored for integrity on a scale of 1–5 by visual examination of their DNA crowns, with samples scoring higher than 2 being excluded from further analyses (see Seeker *et al*. 2016 for details). Samples which failed one or more of the above QC measures were re‐extracted, and if they failed QC a second time, they were excluded from the study.

Relative leucocyte telomere length (RTL) was measured using real‐time quantitative PCR (qPCR; Cawthon 2002), using protocols we have previously developed and validated in sheep and cattle blood samples (Fairlie *et al*. 2016; Seeker *et al*. 2016). The qPCR method estimates the total amount of telomeric sequence present in a sample relative to the amount of a nonvariable copy number reference gene. Note that this method measures both terminal and interstitial telomere sequence, and if interstitial telomeric DNA content varies among or within individuals, this could influence our results. In this study, we used the beta‐2‐microglobulin (B2M) as our reference gene and used primers supplied by Primer Design (Catalogue number: HK‐SY‐Sh‐900, Southampton, UK). For telomeric amplification, tel1b (5′‐CGG TTT GTT TGG GTT TGG GTT TGG GTT TGG GTT TGG GTT‐3′) and tel 2b (5′‐GGC TTG CCT TAC CCT TAC CCT TAC CCT TAC CCT TAC CCT‐3′) primers were used (Epel *et al*. 2004). Telomere primers were manufactured, HPLC‐purified and supplied by Integrated DNA Technologies (IDT, Glasgow, UK). Telomere and reference gene reactions were run in separate wells of the same qPCR plate at a concentration of 300 and 900 nm, respectively. Samples were diluted to 1 ng/μL with buffer AE just prior to qPCR analysis. Each reaction was prepared using 5 μL of LightCycler 480SYBR Green I Master Mix (Cat # 04887352001, Roche, West Sussex, UK) and 1 ng of sample DNA in a total reaction volume of 10 μL. We used 384‐well plates which were loaded with sample DNA and master mix using an automated liquid handling robot (Freedom Evo‐2 150; Tecan).

Each plate included two calibrator samples (1 ng/μL) to account for plate‐to‐plate variation and a nontemplate control (NTC) consisting of nuclease‐free water. The calibrator sample was extracted from a large quantity of buffy coat prepared from blood supplied from a single domestic sheep (Cat# SHP‐BUFCT‐LIHP, Sera Laboratories International LTD, West Sussex, UK). We carried out a large number of extractions from this sample, applied the same quality control as above and then pooled the extracts and aliquoted them for subsequent use. Samples, calibrators and NTCs were all run in triplicate. All qPCRs were performed using a Roche LC480 instrument using the following reaction protocol: 10 min at 95 °C (enzyme activation), followed by 50 cycles of 15 s at 95 °C (denaturation) and 30 s at 58 °C (primer annealing), then 30 s at 72 °C (signal acquisition). Melting curve protocol was 1 min at 95 °C, followed by 30 s at 58 °C, then 0.11 °C/s to 95 °C followed by 10 s at 40 °C.

We used the linregpcr software package (version 2016.0; Ruijter *et al*. 2009) to correct our amplification curves for baseline fluorescence and to calculate well‐specific reaction efficiencies and Cq values. A constant fluorescence threshold was set within the window of linearity for each amplicon group, calculated using the average Cq across the first six plates. The threshold values used were 0.193 and 0.222, and the average efficiency across all plates was 1.88 and 1.91 for the B2M and telomere amplicon groups, respectively. Samples were excluded from further analysis if the coefficient of variation (CV) across triplicate Cq values for either amplicon was >5%, or if at least one of their triplicate reactions had an efficiency that was 5% higher or lower than the mean efficiency across all wells on that plate for the respective amplicon. Overall, nine samples were excluded based on quality control failure at either extraction or qPCR stages, leaving 492 samples available for use in further analyses.

Relative LTL for each sample was calculated, following Pfaffl 2001, using average reaction efficiencies for each plate and Cq for each sample determined by linregpcr as follows:

$$\begin{array}{c} \text{RTL}=(E_{\mathrm{TEL}}^{\left(\mathrm{CqTEL}[\mathrm{Calibrator}]-\mathrm{CqTEL}[\mathrm{Sample}]\right)})/\\ \left(E_{B2M}^{\left(\mathrm{CqB}2M[\mathrm{Calibrator}]-\mathrm{CqB}2M[\mathrm{Sample}]\right)}\right) \end{array}$$

where E_TEL_ and E_B2M_ are the mean reaction efficiencies for the respective amplicon group across all samples on a given plate; CqTEL[Calibrator] and CqB2M[Calibrator] are the average Cqs for the relevant amplicon across all calibrator samples on the plate; and CqTEL[Sample] and CqB2M[Sample] are the average of the triplicate Cqs for the sample for each amplicon.

### Data analysis

We began by checking the distribution of our telomere, FEC and leucocyte proportions (GLR, CD4:CD8 ratio, proportion of CD4 and CD8 T cells that were naïve) data. RTL was normally distributed, but the other variables showed right skew. Log transformation yielded approximately normally distributions, and so log‐transformed FEC and leucocyte proportions were used in all analyses that followed. Eleven samples came from animals that were not caught at birth and had uncertain ages, so they were excluded from further analyses. We then calculated the Pearson's correlation coefficient among RTL and the leucocyte proportion measures. To test how RTL varied with age and sex, we ran linear mixed‐effects models (LMMs) of RTL including individual identity (481 samples from 395 individuals) and qPCR plate (nine plates) as random effects and year, sex and age (as a linear and quadratic covariate) and the interaction between the age terms and sex as fixed effects. We assessed the significance of each term using likelihood ratio tests (LRTs). We subsequently tested whether independent effects of August weight and FEC on RTL were present, having accounted for age effects, by separately adding weight or FEC and their interactions with sex to the model and testing whether the addition improved model fit using LRTs. We further tested whether horn type was associated with RTL by adding a three‐way interaction among age, sex and horn type and all associated lower‐order interactions and sequentially deleting terms until only significant terms remained in the model.

To test our hypothesis that costs of investment in a key secondary sexual trait in males, horn length, might be reflected in RTL, we ran a separate set of LMMs restricting our data set to only normal horned males (*N* = 130). Only a small proportion of Soay ewes have normal horns and exhibit horn growth (74 of 348 females in our data set) and this combined with the absence of evidence that horn growth is costly or important in female reproduction leads us to restrict our analyses to male horn growth. As horns are grown incrementally each year in sheep, horn length is very strongly determined by an animal's age. To avoid the potentially confounding association between age and horn length in our models (as RTL is age dependent in males, see Results), we ran a separate models for lambs (aged < 1 year) and adults (aged 1 year or more). In lambs, horn length reflects horn growth over the first 4 months of life, and we tested its significance by adding both a linear and quadratic horn length fixed effect to a LMM of RTL with plate as random effect (there was only one observation per individual in this data set), alongside year and lamb age in days (as not all lambs were exactly the same age when caught in August) as additional fixed effects. In adult males, horn length inevitably increases with age as horn increments are grown. We tested the association between horn length and RTL in adults in a similar fashion to lambs, but included age in years as a fixed effect to account for the age‐related change in both RTL and horn length.

To test for associations between RTL and leucocyte proportions and determine whether changes in leucocyte cell structure could explain observed patterns of variation in RTL with age and sex, we added all four leucocyte proportion measures into our final overall LMM of RTL and assessed whether previously observed sex and age effects remained significant in this model. We used a similar approach in our models including horn length restricted to normal horned males. For descriptive purposes, we calculated means and standard errors for all immune cell percentages and counts and ratios derived from them both overall and within each major age class (lamb, yearling, adult: 2–6 years, geriatric: >6 years). We then ran LMMs of each immune cell measurement including age, sex and their interaction and year as fixed effects and individual identity as a random effect, and simplified these models as described above. All analyses were conducted in r version 3.2.3 (R Core Team 2012).

## Results

**Figure 1:**
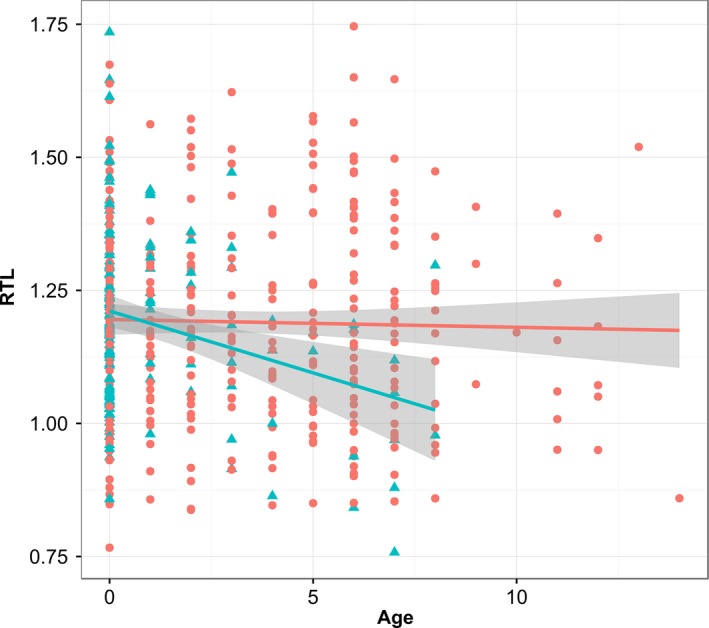
Older males have shorter relative leucocyte telomere length (RTL) than females, but no sex differences are apparent among young animals. Raw data for males (blue triangles) and females (red circles) are presented with a linear regression (blue and red lines, respectively) and associated standard error (grey shading) for each sex. [Colour figure can be viewed at wileyonlinelibrary.com]

There was a significant interaction between the effects of age and sex on RTL ($\chi_{\left(d.f.=1\right)}^{2}$ = 5.67, *P* = 0.02): males, but not females, showed a decline in telomere length with age (estimated difference in slope between males and females: b = −0.019 ± 0.008 SE; Fig. 1). There was no evidence for significant quadratic effects of age (dropping age^2^ and age^2^‐by‐sex interaction from the model: $\chi_{\left(2\right)}^{2}$ = 1.78, *P* = 0.41) and no significant difference in RTL between capture years (dropping year: $\chi_{\left(1\right)}^{2}$ = 1.58, *P* = 0.21). When we separated our RTL data set into age groups and reran our LMMs (without individual as a random effect, because there were no or very few repeat measures in each data subset), we found that male RTL was only significantly shorter than female RTL in adults aged 3 years or more (*N* = 193; males vs. females: b = −0.089 ± 0.042 SE, $\chi_{\left(1\right)}^{2}$ = 4.58, *P* = 0.03). There was no significant sex difference among lambs (*N* = 200; b = 0.004 ± 0.023 SE, $\chi_{\left(1\right)}^{2}$ = 0.32, *P* = 0.86) or individuals aged 2 years or less (*N* = 288; b = 0.016 ± 0.020 SE, $\chi_{\left(1\right)}^{2}$ = 0.68, *P* = 0.43). August weight, FEC and horn type were not significantly associated with RTL independent of age and sex. The addition of weight and its interaction with sex to a model already including an interaction between age and sex did not significantly improve model fit ($\chi_{\left(2\right)}^{2}$ = 1.29, *P* = 0.53), and nor did the addition of FEC and its interaction with sex ($\chi_{\left(2\right)}^{2}$ = 0.36, *P* = 0.84). The addition of horn type and its potential interactions with age and sex also did not significantly improve model fit when added to the model including the interaction between age and sex ($\chi_{\left(6\right)}^{2}$ = 3.12, *P* = 0.79).

**Figure 2:**
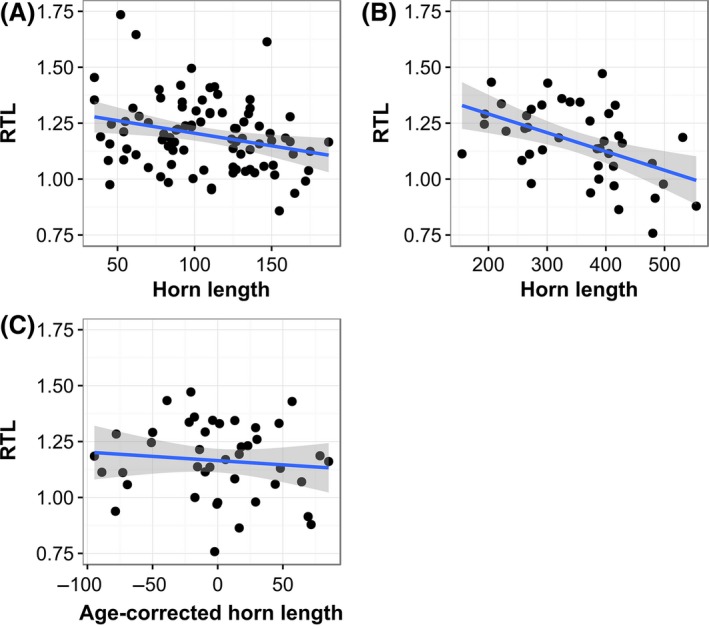
Relative leucocyte telomere length (RTL) is negatively associated with horn length among normal horned males in lambs (<1 year old), but not adults (1 year old or greater) once effects of age are accounted for. Plots show raw RTL against horn length measures with linear regression (black line) and associated standard error (grey shading) for: (A) male lambs, (B) adult males, (C) adult males, having corrected horn length for age by taking residuals from a regression of RTL on age. [Colour figure can be viewed at wileyonlinelibrary.com]

Horn length was negatively associated with RTL in normal horned male lambs, but not in older males once their age was accounted for (Fig. 2). In a model of RTL including only normal horned males, there was a significant decline in RTL with age (b = −0.023 ± 0.008 SE, $\chi_{\left(1\right)}^{2}$ = 9.28, *P* < 0.01). In a model restricted to lambs only, there was no effect of the lamb's age in days at capture in August ($\chi_{\left(1\right)}^{2}$ = 0.35, *P* = 0.55) and a quadratic effect of horn length was not significant ($\chi_{\left(1\right)}^{2}$ = 0.62, *P* = 0.43).

Horn length was significantly negatively associated with RTL (*N* = 88; b = −0.0010 ± 0.0004 SE, $\chi_{\left(1\right)}^{2}$ = 5.43, *P* < 0.05; Fig. 2A). This association remained significant, and actually became stronger, when lamb August weight was included in the model (b = −0.0018 ± 0.0005 SE, $\chi_{\left(1\right)}^{2}$ = 11.56, *P* < 0.001). In models including adult normal horned males, horn length was significant when age was not included in the model (*N* = 42; b = −0.0007 ± 0.0002 SE, $\chi_{\left(1\right)}^{2}$ = 10.06, *P* < 0.01; Fig. 2B), but was nonsignificant when the age was included in the model (b = −0.0002 ± 0.0003 SE, $\chi_{\left(1\right)}^{2}$ = 0.59, *P* = 0.44; Fig. 2C).

**Figure 3:**
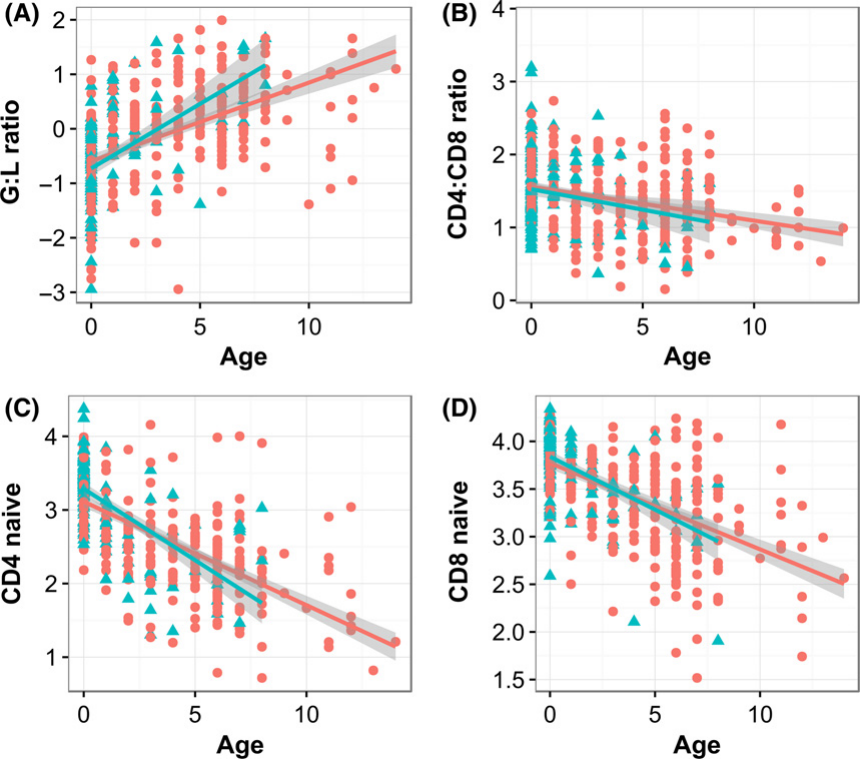
Scatter plots showing relationships with age and sex for (A): granulocyte‐to‐lymphocyte ratios, (B): CD4 T‐cell‐to‐CD8 T‐cell ratios, (C) the proportion of CD4+ ‘helper’ T cells that were naïve and (D) the proportion of CD8+ ‘cytotoxic’ T cells that were naïve. All proportions and ratios are log‐transformed. Raw data for males (blue triangles) and females (red circles) are presented with a linear regression (blue and red lines) and associated standard error (grey shading) for each sex. [Colour figure can be viewed at wileyonlinelibrary.com]

**Figure 4:**
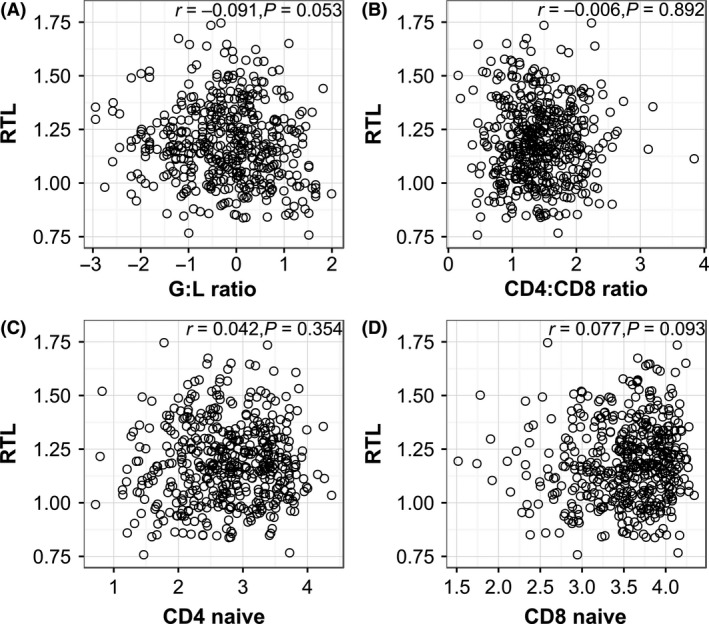
Scatter plots illustrating correlations among relative leucocyte telomere length (RTL) and measures of the proportion of different leucocyte cell types. Scatter plots show relationships between RTL and: (A) granulocyte‐to‐lymphocyte ratio (‘G:L ratio’), (B) CD4+ T‐cell‐to‐CD8+ T‐cell ratio (‘CD4:CD8 ratio’), (C) proportion of CD4+ T cells positive for CD45RA (‘CD4 naïve’) (D) proportion of CD8+ T cells positive for CD45RA (‘CD8 naïve’). All leucocyte ratios and proportions were log‐transformed; Pearson's correlation coefficients and associated *P* values presented for each plot.

Means and standard errors for all immune cell measurements are presented in Table S2 (Supporting information). All four measures of leucocyte proportions used in analyses (GLR, CD4:CD8 ratio and the proportion of naïve CD4 and CD8 T cells) were significantly associated with age: GLR increased with age and the other three measures declined (Fig. 3, Table S3, Supporting information). GLR and the proportion of CD4+ naïve T cells showed significant age‐by‐sex interactions: males increased their GLR and decreased their proportion of CD4 naïve T cells more rapidly with age than females (Fig. 3, Table S3, Supporting information). Variation in RTL and its association with sex and age were largely independent of variation in the proportions of different leucocyte cell types measured in the samples. Pearson's correlation coefficients among RTL and the ratios and proportions of leucocyte cell types ranged between +0.1 and −0.1 (Fig. 4). In a LMM of RTL restricted to samples for which all leucocyte proportion measures were available (*N* = 437), the age‐by‐sex interaction remained marginally nonsignificant (male vs. female slope: b = −0.016 ± 0.009 SE, $\chi_{\left(1\right)}^{2}$ = 3.46, *P* = 0.06). Addition of all four leucocyte proportion measurements to the model of RTL did not improve model fit ($\chi_{\left(4\right)}^{2}$ = 3.34, *P* = 0.50) or meaningfully alter the effect sizes or significance of the age‐by‐sex interaction (b = −0.015 ± 0.009 SE, $\chi_{\left(1\right)}^{2}$ = 3.16, *P* = 0.08). The significant negative association between RTL and horn length in male lambs remained when all four leucocyte cell type measurements were included in the LMM (*N* = 80; b = −0.0010 ± 0.0005, $\chi_{\left(1\right)}^{2}$ = 4.23, *P* < 0.05) and, as before, the addition of the four leucocyte measurements to the model did not improve fit ($\chi_{\left(4\right)}^{2}$ = 4.18, *P* = 0.38).

## Discussion

This study provides, to our knowledge, the first evidence for sex differences in telomere length from a wild mammal. Differences in LTL between males and females were not detectable before 3 years of age, suggesting that there was no sex difference in LTL at birth in our study system. Longer LTLs in females than in males in adulthood but not early life have also been documented in humans and laboratory rodents, suggesting that these sex differences in LTL may arise as a result of differences in attrition rates through development and early adulthood (Cherif *et al*. 2003; Tarry‐Adkins *et al*. 2006; Gardner *et al*. 2014; Lapham *et al*. 2015). In our study, the presence of shorter LTL in older males compared with females could be due to sex differences in telomere attrition rate or in selective mortality associated with telomere length. Sex differences in selection on erythrocyte telomere length have been documented in wild sand lizards (*Lacerta agilis*; Olsson *et al*. 2011), and winter mortality in Soay sheep on St Kilda is male‐biased at all ages (Clutton‐Brock & Pemberton 2004). Current evidence from wild vertebrates, including a previous study of Soay sheep, points to positive associations among LTL or ELT and either annual survival or longevity and thus selective disappearance of individuals with short telomeres (Bize *et al*. 2009; Salomons *et al*. 2009; Olsson *et al*. 2011; Barrett *et al*. 2013; Beirne *et al*. 2014; Fairlie *et al*. 2016). Although sex differences in telomere attrition rate could explain our results, the presence of stronger selective disappearance of individuals with short telomeres in females than in males could also be responsible. We had insufficient longitudinal repeat samples within our very largely cross‐sectional data set to differentiate these two possibilities. Longitudinal telomere data from both sexes spanning the period from birth to later adulthood are required to fully understand the within‐ and among‐individual processes responsible for sex differences in telomere length.

We found no association between LTL and weight or strongyle FEC in either sex, suggesting sex differences in growth or body size and in infection with gastrointestinal parasites could not explain observed sex differences in LTL in later life. This adds to the general lack of support for the sexual size dimorphism hypothesis from both among‐ and within‐species studies (Barrett & Richardson 2011; Olsson *et al*. 2011; Beirne *et al*. 2014). Previous studies have found associations between microparasite infection status and telomere length (Ilmonen *et al*. 2008; Asghar *et al*. 2015, 2016), whilst our study measured burden with chronically infecting gastrointestinal nematode parasites. It seems plausible that a larger longitudinal study may be required to detect the immune consequences of such highly localized and long‐lasting infections and telomere lengths. Studies in laboratory rats have suggested sex differences in TL emerge around puberty as a direct result of the differential effects of sex hormones on telomere dynamics (Cherif *et al*. 2003; Tarry‐Adkins *et al*. 2006). Soay sheep are sexually mature in their first year, so our data imply that sex differences in LTL emerge several years after puberty, but could still be the result of cumulative telomere eroding effects of testosterone relative to oestrogen. Over and above hormonal causes, males surviving to later adulthood will have experienced the cumulative physiological demands associated with years of rutting which could generate further differences in the rate of telomere attrition compared with females.

We have presented rare evidence of reduced telomere length associated with investment in a reproductive trait under natural conditions. Studies of birds in both laboratory and wild populations have found that experimentally increasing reproductive effort decreased ETL in parents, in the short term at least (Heidinger *et al*. 2012; Reichert *et al*. 2014; Sudyka *et al*. 2014; but see Beaulieu *et al*. 2011; Voillemot *et al*. 2012), whilst a nonmanipulative field study found negative associations between ETL and arrival date and the number of nestlings (Bauch *et al*. 2013). We found a significant negative association between LTL and horn length in males at around 4 months of age, but not in adult males. This is consistent with LTL shortening reflecting some physiological cost of horn growth, but raises the question of why it was only detected in lambs. Lambs aged 4 months are growing rapidly and contending with their first exposures to the parasite fauna on St Kilda (Clutton‐Brock & Pemberton 2004), and this may mean that the costs of investment in secondary sexual traits such as horn growth are most pronounced at this age. This stage also captures the relationship between LTL and horn growth prior to potentially confounding effects of overwinter viability selection and subsequent rutting effort and incremental horn growth, which could make the cost easier to detect. A study of wild sand lizards documented disruptive selection on ETL in females but not in males (Olsson *et al*. 2011), and previous work on Soay sheep showed that the alleles associated with horn growth improves breeding success at a cost to longevity in males (Johnston *et al*. 2013). These studies and our present results suggest that the degree to which sex differences in LTL are generated and maintained by sexual differences in selection on telomere length is an important area for future study.

Our study offers indirect evidence that the observed relationships among LTL, age and sex are not driven by variation in the proportions of particular leucocyte cell types, which are known to have different telomere lengths during adulthood in humans (Weng 2001; Kimura *et al*. 2010; Aubert *et al*. 2012). We documented age‐related changes in the proportions of different leucocyte cell types that were consistent with observations in humans and laboratory mice, and in our own previous studies of this system (Linton & Dorshkind 2004; Pawelec *et al*. 2010; Nussey *et al*. 2012). Of particular note, the decline in the proportion of naïve helper and cytotoxic T cells (Fig. 3) with age could generate population‐level declines in mean LTL because naïve T cells have longer telomeres than effector and memory T cells (Weng 2001; Aubert *et al*. 2012). Furthermore, the presence of a sex‐by‐age interaction for CD4+ naïve cells, indicating that males have more rapid rates of declines with age than females (Fig. 3, Table S3, Supporting information), could have been responsible for the observed sex difference in LTL with age. However, we found little evidence that LTL was correlated with any of the leucocyte proportion measurements, and we showed that the main results of our LTL models were not influenced by the inclusion of the leucocyte proportion measurements. Our remote field study site precluded the use of more sophisticated methods to determine the actual telomere lengths of particular types of leucocytes, such as cell sorting or flow‐FISH (Kimura *et al*. 2010; Aubert *et al*. 2012). However, our findings are consistent with the mounting evidence that TL is highly correlated across leucocyte cell subsets, and across tissues more generally, within individual organisms (Kimura *et al*. 2010; Aubert *et al*. 2012; Daniali *et al*. 2013; Reichert *et al*. 2013; Asghar *et al*. 2016). This suggests that telomere length, whether measured in leucocytes or erythrocytes, could reflect variation that exists in the individual's haematopoietic stem cell compartment, and even more general organism‐wide variation in telomere length (Kimura *et al*. 2010; Daniali *et al*. 2013; Reichert *et al*. 2013).

## Data accessibility

Data used in this manuscript are available on Dryad: https://doi.org/10.5061/dryad.kd92s.

All authors contributed to the design of the study; R.L.W. and D.H.N. wrote the manuscript with editorial input from all coauthors; R.L.W., H.F. and D.H.N. conducted the statistical analyses; R.L.W., E.J.B., J.F., S.U., K.W., E.S.‐C. and R.V.A. conducted the laboratory work; J.M.P. and J.G.P. coordinated field trips and conducted data and sample collection in the field.

## Supporting information


**Appendix S1** Supplementary methods
**Table S1** Details of the reagents used in the flow cytometry analysis of T cell subsets.
**Table S2** Average proportions of different T cell sub‐populations, counts of different leukocyte subsets and ratios calculated from these values for main sheep age groups (adult: 2–6 years, geriatric: >6 years) and overall with standard errors.
**Table S3** Summary of linear mixed models relating log transformed leukocyte cell proportion measures to age and sex.
