# Supplementary material for: Sex differences in leucocyte telomere length in a free‐living mammal
Source: Mol Ecol. 2017 Jan 27;26(12):3230–40. doi: 10.1111/mec.13992 (PMC5484296; doi:10.1111/mec.13992)
Supplement: Supplementary file 1 — Appendix S1 Supplementary methods Table S1 Details of the reagents used in the flow cytometry analysis of T cell subsets. Table S2 Average proportions of different T cell sub‐populations, counts of different leukocyte subsets and ratios calculated from these values for main sheep age groups (adult: 2–6 years, geriatric: >6 years) and overall with standard errors. Table S3 Summary of linear mixed models relating log transformed leukocyte cell proportion measures to age and sex. [file MEC-26-3230-s001.docx]

**Supplementary on-line material for:**

Sex differences in leukocyte telomere length in a wild mammal, by Watson *et al.*

**Supplementary methods:**

Flow cytometry: In the field on St Kilda, using the second of the 9 ml Lithium Heparin vacutainers of blood collected from each animal, red blood cells were lysed by adding 5 ml of ammonium chloride lysing solution (1.5 M NH4Cl, 100 mM NAHCO3, 10 mM Na2EDTA) to 1 ml of whole blood ~~and~~, mixing gently and centrifuging at 1008 x g for 10 minutes. The supernatant was removed and the cell pellet re-suspended in 9 ml of phosphate buffered saline (PBS) to wash. The sample was then centrifuged (as above), the supernatant removed and the pellet re-suspended in 2.5 ml 1 % paraformaldehyde (PFA) in PBS and incubated at room temperature for 10 minutes. The sample was then centrifuged at 1008 x g for 10 minutes, the supernatant was removed and the cell pellet re-suspended in 9 ml phosphate buffered saline (PBS) to wash. The cells were spun again at 1008 x g for 10 minutes and finally re-suspended in PBS + 0.02 % NaN3 solution. The samples were stored at 4 °C until staining and analysis by flow cytometry in Edinburgh.

In the laboratory in Edinburgh, fluorescently labelled monoclonal antibodies were used to identify the proportions of T helper cells (CD4+)and~~,~~ cytotoxic T cells (CD8+). The proportion of naïve helper and naïve cytotoxic T cells, were identified by their co-expression of the CD45RA marker (see Table S1 below). A regulatory cell (Treg) Foxp3 was also measured in this protocol, but this measure was not included in the present analysis. We used a multi-panel stain performed over a two-day protocol. On the first day cells were stained for CD4+, CD8+, and naïve (CD45RA+) cell types and the overnight incubation was set up to make the cell walls permeable in preparation for the intracellular FoxP3 stain. 100 µl of fixed cells was added to each well; one individual per well in sample wells and an aliquot of a generic ‘golden sample’ made up of multiple individuals to control wells. The plates were spun at 2000 rpm for 1 minute at 4 °C, before removing the supernatant by flicking plate downwards into sink, taking care not to lose the pellet. Non-specific binding sites were blocked by re-suspending cells in 200 µl of a 20 % solution of Normal Goat Serum diluted in FACS buffer (PBS + 5 % heat-inactivated foetal calf serum + 0.02 % sodium azide) to each well and incubating at 4 ˚C for 30 minutes. Plates were spun as before and then re-suspended in 100 µl of antibody mix in each sample well, 100 µl of each antibody in the corresponding control well and 100 µl of a specific antibody control mix in the negative control wells (fluorescence minus one (FMO)). Remaining wells were re-suspended in 100 µl FACS buffer and plates were incubated at 4 ˚C for 30 minutes before spinning as before. Plates were washed by adding 100 µl FACS buffer to each well, before spinning and re-suspending in 200 µl FACS buffer for the final wash step. Plates were spun as before and the re-suspended in 200 µl permabilisation solution (PBS + 0.2 % saponin + 20 % normal rat serum) in all wells. Plates were incubated at 4 ˚C overnight. On day two of the protocol, plates were spun as before and either re-suspended in 100 µl anti-FoxP3 647 (1:80) for sample and corresponding control wells or permabilisation buffer without rat serum (PBS + 0.2 % saponin) for the remaining wells. Plates were incubated at 4˚C for one hour and then washed in a two-step process as before. Cells in all wells were re-suspend in 200 µl PBS solution and then plates were covered and stored at 4 °C for less than 24 hours before reading on the MACSQuant flow cytometer (Miltenyi Biotech Ltd).

Flow cytometry data was analysed using FlowJo version X.0.7 analysis software (TreeStar, San Carlos, CA). Proportions of T cells were measured by firstly placing a gate encompassing the entire lymphocyte population. T cell populations were then gated using specific CD4 or CD8 fluorescence, followed by appropriate sub-type gating based on CD45RA fluorescence. Those with poor staining, poor cell profiles or low cell numbers were omitted from analysis leaving an overall number of 449 samples for CD4:CD8 and 446 samples for both naïve cell subtypes.

DNA Extraction: Genomic DNA was extracted from buffy coat using the Qiagen DNeasy Blood and Tissue Kit following manufacturer’s guidelines for animal blood (Cat# 69581, Manchester, UK). The protocol was modified slightly to facilitate sample flow through the spin columns which subsequently improved DNA yield and purity (see SOM). These are outlined below:

1. Prior to step 1a, 50 µl of buffy coat was mixed thoroughly with 300 µl Qiagen Red Blood Cell (RBC) lysis solution (Cat# 158902, Manchester, UK) and then centrifuged for 30 s at 14000 x g to produce a white blood cell (WBC) pellet. The supernatant was then discarded leaving approximately 10 µl residual liquid. 100 µl PBS was added to the sample and the WBC pellet re-suspended by vortexing. This step removes all remaining red blood cells.
2. The volume of proteinase K added in step 1a was increased from 20 µl to 30 µl to ensure complete WBC digestion.
3. Incubation with buffer AL at 56 ^o^C in step 2 was increased from 10 min to 1 hour, vortexing the sample at 30 min mid- incubation, to optimise WBC lysis.

**Table S1**. Details of the reagents used in the flow cytometry analysis of T cell subsets. All antibodies were used directly conjugated and were either sourced as direct conjugates commercially or conjugated using commercial kits with antibodies produced in-house.

| Antibody | Host/isotype* | Clone | Conjugate | Labelling** | Dilution  (µg/ml) | FACSCanto  Excitation  (nm) | sEmission (nm) |
| --- | --- | --- | --- | --- | --- | --- | --- |
| Anti-CD4 | Mouse IgG2a | 17D | Alexa Fluor® 488 | Innova: nlightening system | 0.2 | 488 | 530/30 |
| Anti-CD8 | Mouse anti Sheep IgG2a | LT8 | PE | Commercial | 0.1 | 488 | 575/26 |
| Anti-CD45RA | Mouse IgG1 | 73B | PerCp-Cy5.5 | Lightning-Link® | 0.02 | 488 | 695/40 |

**Table S2.** Average proportions of different T cell sub-populations, counts of different leukocyte subsets and ratios calculated from these values for main sheep age groups (adult: 2-6 years, geriatric: >6 years) and overall with standard errors.

| Age | %CD4 | SE | %CD8 | SE | % CD4 naïve | SE | % CD8 naïve | SE | Lymphocytes | SE | Neutrophil | SE | Eosinophil | SE | Granulocyte:  Lymphocyte | SE | CD4:CD8 | SE |
| --- | --- | --- | --- | --- | --- | --- | --- | --- | --- | --- | --- | --- | --- | --- | --- | --- | --- | --- |
| Lamb | 22.65 | 0.43 | 5.15 | 0.15 | 29.68 | 0.72 | 48.64 | 0.84 | 68.29 | 1.09 | 28.50 | 1.02 | 2.97 | 0.24 | 0.55 | 0.03 | 5.24 | 0.21 |
| Yearling | 26.80 | 0.96 | 5.91 | 0.43 | 18.51 | 1.33 | 38.89 | 1.61 | 51.28 | 2.79 | 41.95 | 2.56 | 6.53 | 0.74 | 1.24 | 0.12 | 5.53 | 0.40 |
| Adult | 28.85 | 0.55 | 8.32 | 0.28 | 12.52 | 0.64 | 31.13 | 0.96 | 45.37 | 1.29 | 47.78 | 1.25 | 6.56 | 0.42 | 1.57 | 0.09 | 4.14 | 0.17 |
| Geriatric | 31.42 | 0.92 | 10.07 | 0.61 | 10.15 | 1.13 | 25.76 | 1.83 | 37.85 | 1.99 | 54.31 | 1.87 | 7.74 | 0.97 | 2.07 | 0.16 | 3.76 | 0.23 |
| **Overall** | **26.45** | **0.34** | **7.02** | **0.17** | **19.77** | **0.57** | **38.28** | **0.71** | **54.32** | **0.94** | **40.20** | **0.85** | **5.25** | **0.25** | **1.19** | **0.05** | **4.67** | **0.12** |

**Table S3.** Summary of linear mixed models relating log transformed leukocyte cell proportion measures to age and sex. Terms in bold were retained in the final model and terms in normal font were sequentially removed due to non-significance. Effect sizes (b +/- SE) are only reported for significant terms retained in the final models. Individual identity was included as a random effect in all models.

|  | Year (2015 vs. 2014) | | Age (female) | | Sex (male vs. female) | | Age by sex (male slope vs. female slope) | |
| --- | --- | --- | --- | --- | --- | --- | --- | --- |
|  | *b* (se) | X^2^ (P value) | *b* (se) | X^2^ (P value) | *b* (se) | X^2^ (P value) | *b* (se) | X^2^ (P value) |
| Granuloctye to lymphocyte ratio | **0.157 (0.075)** | **4.40 (0.036)** | **0.145 (0.014)** |  | **-0.123 (0.101)** |  | **0.096 (0.038)** | **6.39 (0.011)** |
| CD4 to CD8 ratio | **0.468 (0.033)** | **156.6 (<0.001)** | **-0.044 (0.006)** | **47.5 (<0.001)** |  | 0.50 (0.50) |  | 0.00 (0.92) |
| Proportion of CD4+ naïve T cells | **-0.232 (0.032)** | **47.2 (<0.001)** | **-0.143 (0.009)** |  | **0.165 (0.063)** |  | **-0.057 (0.023)** | **6.33 (0.012)** |
| Proportion of CD8+ naïve T cells | **-0.230 (0.032)** | **46.9 (<0.001)** | **-0.096 (0.006)** | **201.3 (<0.001)** |  | 0.40 (0.55) |  | 1.50 (0.22) |
